# Characterization of the SARS-CoV-2 Host Response in Primary Human Airway Epithelial Cells from Aged Individuals

**DOI:** 10.3390/v13081603

**Published:** 2021-08-12

**Authors:** Bharathiraja Subramaniyan, Jason L. Larabee, Manish Bodas, Andrew R. Moore, Anthony W. G. Burgett, Dean A. Myers, Constantin Georgescu, Jonathan D. Wren, James F. Papin, Matthew S. Walters

**Affiliations:** 1 Department of Medicine, Section of Pulmonary, Critical Care & Sleep Medicine, University of Oklahoma Health Sciences Center, Oklahoma City, OK 73104, USA; Bharathiraja-Subramaniyan@ouhsc.edu (B.S.); manish-bodas@ouhsc.edu (M.B.); Andrew-R-Moore@ouhsc.edu (A.R.M.); 2 Department of Microbiology and Immunology, University of Oklahoma Health Sciences Center, Oklahoma City, OK 73104, USA; Jason-Larabee@ouhsc.edu; 3 Department of Pharmaceutical Sciences, University of Oklahoma Health Sciences Center, Oklahoma City, OK 73104, USA; Anthony-Burgett@ouhsc.edu; 4 Department of Obstetrics and Gynecology, University of Oklahoma Health Sciences Center, Oklahoma City, OK 73104, USA; Dean-Myers@ouhsc.edu; 5 Genes & Human Disease Research Program, Oklahoma Medical Research Foundation, Oklahoma City, OK 73104, USA; Constantin-Georgescu@omrf.org (C.G.); Jonathan-Wren@omrf.org (J.D.W.); 6 Department of Pathology, Division of Comparative Medicine, University of Oklahoma Health Sciences Center, Oklahoma City, OK 73104, USA; James-Papin@ouhsc.edu

**Keywords:** COVID-19, SARS-CoV-2, air–liquid interface, airway epithelium, immune response, innate immunity, inflammation, aging

## Abstract

Severe Acute Respiratory Syndrome Coronavirus 2 (SARS-CoV-2) is the causative agent of coronavirus disease 2019 (COVID-19), a global pandemic characterized by an exaggerated immune response and respiratory illness. Age (>60 years) is a significant risk factor for developing severe COVID-19. To better understand the host response of the aged airway epithelium to SARS-CoV-2 infection, we performed an in vitro study using primary human bronchial epithelial cells from donors >67 years of age differentiated on an air–liquid interface culture. We demonstrate that SARS-CoV-2 infection leads to early induction of a proinflammatory response and a delayed interferon response. In addition, we observed changes in the genes and pathways associated with cell death and senescence throughout infection. In summary, our study provides new and important insights into the temporal kinetics of the airway epithelial innate immune response to SARS-CoV-2 in older individuals.

## 1. Introduction

Coronavirus disease 2019 (COVID-19) is a global pandemic caused in response to infection with Severe Acute Respiratory Syndrome Coronavirus 2 (SARS-CoV-2), a highly transmissible and pathogenic virus [1,2]. To date, more than 120 million cases have been confirmed worldwide, including over 3.5 million deaths [3]. SARS-CoV-2 is an enveloped, positive-sense, single-stranded RNA virus that is a member of the Betacoronavirus family that also includes the pathogenic coronaviruses SARS-CoV-1 and Middle East Respiratory Syndrome Coronavirus (MERS) [1]. The genome of SARS-CoV-2 is ~30 kb in size and is predicted to encode 16 nonstructural proteins (nsp1-16); 8 accessory proteins (3a, 3b, 6, 7a, 7b, 8b, 9b and 14) and four structural proteins (spike, membrane, envelope and nucleocapsid) [1,4]. The human respiratory tract is the primary route of SARS-CoV-2 infection [1,5,6,7,8]. During the past year, in vitro organotypic cell cultures of the human airway epithelium have been used extensively to help further our knowledge of SARS-CoV-2 biology and the host response to infection [9]. These include air–liquid interface (ALI) culture systems that support the differentiation of primary human bronchial epithelial cells (HBECs) into a mucociliary epithelium and alveosphere cultures of distal lung alveolar type 2 cells that mimic key aspects of the in vivo epithelium present in the upper airways and distal lung, respectively [5,6,10,11,12,13,14,15,16,17,18,19,20,21,22,23,24,25,26]. These studies have helped demonstrate that virus entry is mediated by binding of the viral spike protein to the host receptor angiotensin converting enzyme II (ACE2) located on the surface of respiratory epithelial cells in the upper airways (e.g., ciliated cells) and distal lung (e.g., alveolar type 2 cells) [5,7,8,27,28,29,30,31,32]. Upon binding to ACE2, proteolytic priming of the spike protein via cellular proteases (e.g., TMPRSS2 and FURIN) triggers the fusion of the viral envelope with the cell membrane, leading to the release of the viral genome into the host cell [1,27,28,30]. Following virus replication, the newly formed infectious virus spreads within the airways and to the distal alveoli [5].

The clinical outcome of SARS-CoV-2 infection varies from patient to patient and is characterized by a variable presentation of symptoms, including, fever, cough, shortness of breath, sore throat and general malaise [2,33]. While the majority of patients are asymptomatic or experience a mild form of the disease, severe cases of COVID-19 can lead to acute respiratory distress syndrome (ARDS) and death [2,33]. The variability in disease severity between patients has been linked to differences in the host inflammatory response to the virus infection, with severe COVID-19 patients having increased levels of proinflammatory cytokines (e.g., IL-6 and TNFα) and chemokines (e.g., CCL2 and CXCL10) compared to patients with a mild level of the disease [10,34,35,36,37,38,39]. These data suggest that an exaggerated host immune response (i.e., “cytokine storm”) to SARS-CoV-2 infection plays a critical role in regulating the disease progression in patients with severe COVID-19. However, the underlying host factors and mechanisms that regulate this immune response following SARS-CoV-2 infection remain unclear.

The major risk factors for the development of severe COVID-19 include cigarette smoking, obesity, cardiovascular disease and diabetes [40,41,42,43,44]. Furthermore, higher mortality rates are observed in men compared to women [42]. Additionally, there is strong evidence that age is the most significant risk factor, with individuals > 60 years of age showing the highest rates of morbidity and mortality [42,45,46]. To better understand the temporal kinetics of the host response of this demographic group to SARS-CoV-2 infection, we performed an in vitro-based study using primary HBECs from donors > 67 years of age. The HBECs from each donor were differentiated into a mucociliary epithelium on an in vitro ALI culture and then infected with SARS-CoV-2 to study the virus replication and the host response using both targeted (i.e., qPCR) and genome-wide (i.e., bulk RNA-Seq) approaches throughout the course of infection. Our results demonstrated that ALI differentiated epithelia generated from aged HBEC donors are permissive to SARS-CoV-2 infection, resulting in the production of an infectious virus and a potent host response. Virus infection leads to the induction of a proinflammatory response at the early stages of infection and a delayed interferon (IFN) response. Furthermore, virus infection leads to changes in the genes and pathways associated with cell death and senescence. In summary, our study provides new and important insights into the dynamics of the virus–host response of SARS-CoV-2 in airway epithelial cells from aged individuals.

## 2. Materials and Methods

### 2.1. Generation and Titration of Severe Acute Respiratory Syndrome-Related Coronavirus 2 (SARS-CoV-2) Stocks

The SARS-CoV-2 isolate USA-WA1/2020 was deposited by the Centers for Disease Control (CDC) and Prevention and obtained through BEI Resources, NIAID, NIH (catalog number NR-52281, Manassas, VA, USA). Viral stocks were produced following 3 passages in Vero E6 cells (catalog number CRL-1586, ATCC, Manassas, VA, USA) cultured in Dulbecco’s Modified Eagle’s Medium (DMEM) (catalog number 11885092, Thermo Fisher Scientific, Waltham, MA, USA) supplemented with 5% fetal bovine serum (FBS) (catalog number 35-011-CV, Corning^®^, Corning, NY, USA) and penicillin (100 I.U./mL)–streptomycin (100 µg/mL) and maintained at 37 °C with 5% CO_2_. To passage SARS-CoV-2, Vero E6 cells were grown to 50% confluence (~1 × 10^7^ cells) in a T-150cm^2^ flask and inoculated with SARS-CoV-2 at a multiplicity of infection (MOI) of 0.001. Forty-eight hours post-infection, the supernatants from the infected cells were harvested, aliquoted and stored at −80 °C. Virus stocks were titrated using the 50% tissue culture infectious dose (TCID_50_) method. Briefly, the Vero E6 cells were seeded in a 96-well plate (1 × 10^4^ cells per well) immediately before the assay and then infected with 10-fold serial dilutions (10^−1^ to 10^−6^) of the SARS-CoV-2 virus stocks. At 96 h post-infection, each well was visually inspected under a microscope to determine which wells were positive for SARS-CoV-2-induced cytopathic effects (CPE). At each dilution, the number of CPE positive wells was recorded and used to calculate the virus titer (TCID_50_/mL) using the Reed and Muench method, as previously described [47]. The conversion of TCID_50_/mL to mean plaque-forming units per mL (PFU/mL) was performed by multiplying the TCID50/mL titer by a factor of 0.7 (https://www.atcc.org/support/technical-support/faqs/converting-tcid-50-to-plaque-forming-units-pfu, accessed on 20 May 2021). All the experiments involving SARS-CoV-2 were conducted using a virus from the same viral stock and were performed in the High Containment Biosafety Level-3 Laboratory Core at the University of Oklahoma Health Sciences Center, Oklahoma City, OK, USA under the Institutional Biosafety Committee approved biosafety protocol #100492.

### 2.2. Human Bronchial Epithelial Cell (HBEC) Cultures and Air–Liquid Interface (ALI) Culture

Primary HBECs isolated from the trachea–bronchial region of normal nonsmokers were purchased commercially (catalog number CC-2540, Lonza, Morristown, NJ, USA) and maintained as previously described [48]. Cells from *n* = 4 donors >67 years of age were used in this study with the following lot numbers and demographics: Donor 1 (lot number: 0000529235, female, 67 years old), Donor 2 (lot number: 0000444771, male, 69 years old), Donor 3 (lot number: 0000608196, male, 67 years old) and Donor 4 (lot number: 0000420927, female, 69 years old). HBECs were differentiated using the ALI culture for 29–31 days to generate a mucociliary airway epithelium, as described previously [48]. All cell culture experiments were performed at 37 °C with 5% CO_2._

### 2.3. Infection of ALI Cultures

Differentiated ALI cultures were infected with SARS-CoV-2 (average of 1.45 × 10^4^ PFU per ALI well) via the apical chamber of the Transwell insert at a MOI of 0.1 calculated based on the total number of cells per ALI well at the time of infection. The cells were either mock-infected (uninfected) or infected with SARS-CoV-2 in the Human Bronchial/Tracheal Epithelial Cell (HBTEC) ALI differentiation medium (catalog number LM-0050, Lifeline^®^ Cell Technology, Oceanside, CA, USA) by adding 100 μL of inoculum to the apical chamber. The mock-infected cells received an equal volume of media used for the SARS-CoV-2 inoculum. After a 2 h of incubation at 37 °C with 5% CO_2_, the inocula were removed from the apical chamber to expose the cells to the air for the remainder of the experiment. At each time point post-infection (24–96 h), the medium from the basolateral chamber of the mock- or SARS-CoV-2-infected cells was collected and stored at −80 °C, with the RNA extracted from the remaining cells as described below. Furthermore, the cells from a single SARS-CoV-2-infected ALI well were frozen at −80 °C in the presence of 100-μL HBTEC media to quantify the virus production by the TCID_50_ assay. The apical chamber media samples were later thawed and subjected to 10-fold serial dilutions (10^−1^ to 10^−6^) and inoculated onto Vero E6 cells as described above for the TCID_50_ assays.

### 2.4. RNA Extraction and cDNA Synthesis

Total RNA was extracted via the direct lysis of cells in the ALI well using the PureLink^TM^ RNA mini kit (catalog number 12183018A, Thermo Fisher Scientific) and included DNase treatment (catalog number 12185-010, Thermo Fisher Scientific) on the column to remove the contaminating genomic DNA. Complementary DNA (cDNA) was generated from an equal amount of total RNA per sample for each donor using random hexamers (Applied Biosystems^TM^ High-Capacity cDNA Reverse Transcription Kit, catalog number 4374966, Thermo Fisher Scientific).

### 2.5. Gene Expression Analysis

Quantitative PCR (qPCR) was performed using the iTaq^TM^ Universal SYBR^®^ Green supermix (catalog number 1725124, Bio-Rad, Hercules, CA, USA) on the Bio-Rad CFX96 Touch^TM^ Real-Time PCR system. The relative expression levels of specific genes were analyzed in duplicate and determined using the dCt method with Actin Beta (ACTB) as the endogenous control. The following PrimePCR^TM^ gene-specific primers were purchased from Bio-Rad: ACTB (qHsaCED0036269), KRT5 (qHsaCED0047798), SCGB1A1 (qHsaCID0018013), MUC5AC (qHsaCID0017663), DNAI1 (qHsaCID0017936), ACE2 (qHsaCID0009100), TMPRSS2 (qHsaCID0013558), FURIN (qHsaCED0044970), CCL2 (qHsaCID0011608), CCL20 (qHsaCID0011773), CXCL6 (qHsaCED0002472), IL-6 (qHsaCID0020314), TNFα (qHsaCED0037461), INFβ1 (qHsaCED0019234), CXCL10 (qHsaCED0046619), ISG15 (qHsaCED0001967), MX1 (qHsaCED0045780), MX2 (qHsaCID0014994) and the assays performed using the manufacturer’s recommended cycling parameters. Expression of the SARS-CoV-2 nucleocapsid gene was quantified using the CDC-designed primers nCOV_N1 Forward Primer (catalog number 10006821) and nCOV_N1 Reverse Primer (catalog number 10006822) purchased from IDT (San Diego, CA, USA) and the following cycling parameters: 25 °C for 2 min, 50 °C for 15 min, 95 °C for 2 min and 45 cycles of 95 °C for 3 s and 55 °C for 30 s. The final concentration of the primers in each reaction was fixed to 500 nM, and a plasmid containing the SARS-CoV-2 nucleocapsid gene (catalog number 10006625, IDT) was used as a positive control. For each time point and condition, the gene expression levels were assessed in *n* = 3 ALI wells with the means used for the statistics.

### 2.6. Bulk RNA Sequencing (Bulk RNA-Seq)

Uninfected or SARS-CoV-2 infected cells (*n* = 4 donors) were harvested as a function of time post-infection (24–96 h) and the genome-wide transcriptome changes in response to the SARS-CoV-2 infection assessed by the bulk RNA-Seq. The libraries were prepared using the QuantSeq 3′ mRNA-Seq Library Prep Kit FWD from Illumina (Lexogen, Vienna, Austria) with the total RNA used as the input. The sequencing of each library was performed on a NextSeq 500 Flowcell, High SR75 (Illumina, San Diego, CA, USA). The raw sequencing reads (in a FASTQ format) were trimmed of the residual adaptor sequences using Scythe software, and the low-quality bases at the beginning or end of the sequencing reads were removed using sickle. The quality of the remaining reads was confirmed with the FastQC utility. The trimmed sequencing reads were aligned to the *Homo sapiens* genome reference (GRCh38/hg38) using STAR v2.4.0h [49]. The gene-level read counts were determined using HTSeq v0.5.3p9 [50] with the GENCODE Release 38 (GRCh38.p13) annotation. The read count normalization and differentially expressed analyses were performed using the edgeR package from Bioconductor, following the widely used limma/voom workflow [51]. Proper modeling of the repeated measurements over several time points and treatments of the same patient cultured cells was achieved in the context of linear mixed models using the DREAM methodology [52]. Genes that were significantly differentially expressed in response to SARS-CoV-2 infection at each time point were determined using a threshold *p*-value of 0.01 (*p* < 0.01). The Ingenuity Pathway Analysis (IPA) (Qiagen, Redwood City, CA, USA) was used to identify the molecular pathways altered in response to the virus infection. The volcano plots and heatmaps were generated using R. The raw data from the bulk RNA-Seq studies are publicly available at the Gene Expression Omnibus (GEO) site (http://www.ncbi.nlm.nih.gov/geo/), accession number GSE175779.

### 2.7. Immunofluorescence Staining

The immunofluorescent staining of SARS-CoV-2-infected cells in fixed ALI wells was performed as described [48] using a primary antibody specific for the SARS-CoV-2 nucleocapsid (10 µg/mL, catalog number MA1-7403, Thermo Fisher Scientific) and P21 (CDKNA1) (0.61 µg/mL, catalog number 2947, Cell Signaling Technology, Danvers, MA, USA). Isotype-matched mouse IgG (catalog number 401402, BioLegend, San Diego, CA, USA) and rabbit IgG (catalog number 02-6102, Thermo Fisher Scientific) were used as the negative controls. To visualize the antibody binding, goat anti-mouse Alexa Fluor 488 (2 µg/mL, catalog number A11029, Thermo Fisher Scientific) and goat anti-rabbit Alexa Fluor 546 (2 µg/mL, catalog number A11035, Thermo Fisher Scientific) were used as a secondary antibody, and the cell nuclei were counterstained with DAPI (1 µg/mL, catalog number 62248, Thermo Fisher Scientific). The images were taken using an Olympus BX43 upright fluorescent microscope (Olympus America Inc., Waltham, MA, USA). For quantification of the SARS-CoV-2 nucleocapsid and P21 (CDKNA1)-positive cells, a minimum of *n* = 5 random images of the epithelium were taken for each culture, and a minimum of 1200 cells were counted. The number of nucleocapsid and P21 (CDKNA1)-positive cells were counted using ImageJ software (version 1.8.0_112, NIH) and normalized to the number of nuclei.

### 2.8. Statistics

The statistical analysis of the comparisons between SARS-CoV-2-infected cultures vs. uninfected controls was performed using SPSS Version 27.0 software (SPSS Inc., Chicago, IL, USA). The statistical significance was determined as a *p*-value of ≤ 0.05 using the Mann–Whitney *U* test.

## 3. Results

### 3.1. HBECs from Aged Individuals Are Permissive to SARS-CoV-2 Infection

**Figure 1:**
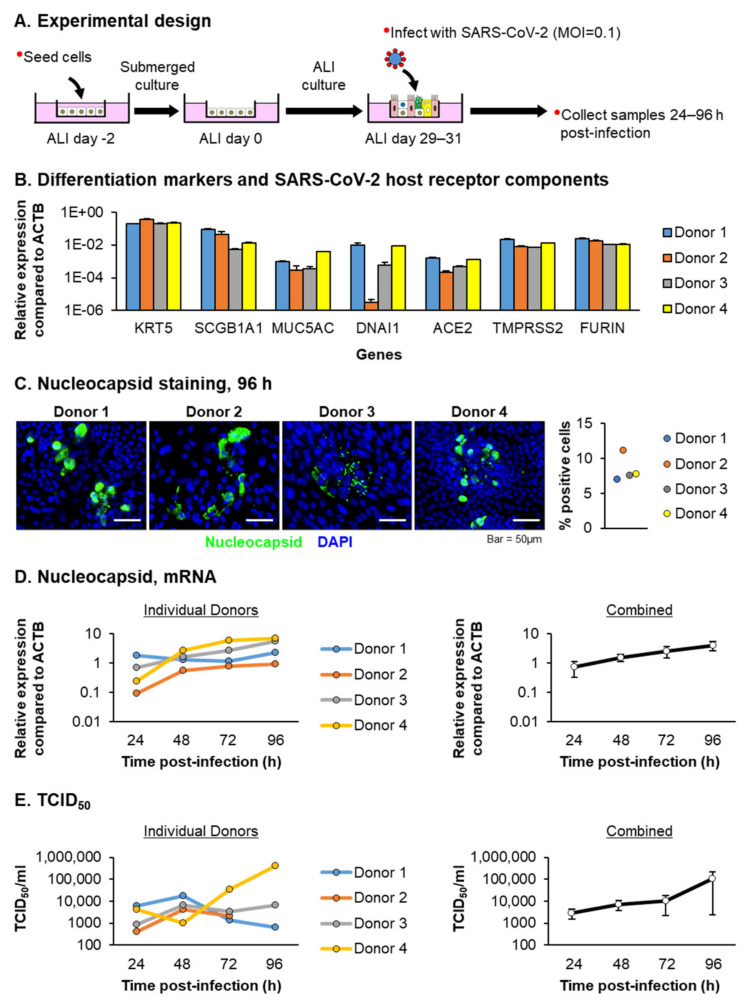
Primary HBEC cultures from aged donors are permissive to SARS-CoV-2 infection. (**A**) Experimental design. Primary HBECs (*n* = 4 donors) were cultured on ALI for 29–31 days and then infected with SARS-CoV-2 at a MOI of 0.1. The cells were then harvested as a function of time post-infection (24–96 h) for an analysis of the viral replication. (**B**) qPCR analysis of the differentiation markers (KRT5 (basal cell), SCGB1A1 (club cell), MUC5AC (goblet cell) and DNAI1 (ciliated cell)) and the SARS-CoV-2 host receptor component genes (ACE2, TMPRSS2 and FURIN) in each donor at the time of infection. The data are presented as the mean relative expression compared to ACTB from *n* = 3 ALI wells for each donor. Error bars indicate the SEM. (**C**) Immunofluorescent staining of SARS-CoV-2 nucleocapsid (green) and the nuclei (blue, DAPI) in cultures 96 h post-infection for each donor. Scale bar = 50 µm. Quantification of the percentage of nucleocapsid-positive cells in each donor at 96 h post-infection. (**D**) qPCR analysis of the SARS-CoV-2 nucleocapsid gene expression. In the left panel, the data are presented for each individual donor, with each data point representing the mean relative expression from *n* = 3 ALI wells. In the right panel, the data are combined and presented as the mean relative expression from *n* = 4 donors. The error bars indicate the SEM. (**E**) Fifty percent tissue culture of an infectious dose (TCID_50_) of the virus. In the left panel, the data are presented for each individual donor, with each data point representing the TCID_50_/mL calculated from a single ALI well. In the right panel, the data are combined and presented as the mean TCID_50_/mL from *n* = 4 donors. The error bars indicate the SEM.

The mucociliary epithelium of the upper human respiratory tract is likely the primary site of SARS-CoV-2 infection in vivo [1,5,6,7,8]. Therefore, we utilized the in vitro ALI culture model to study the virus replication kinetics and the host response following SARS-CoV-2 infection of a mucociliary epithelium generated using primary HBECs isolated from older individuals (>67 years old). The cells were cultured at ALI for 29–31 days (Figure 1A), and the differentiation of each HBEC donor was confirmed by the qPCR analysis for specific markers of basal (KRT5), club (SCGB1A1), goblet (MUC5AC) and ciliated (DNAI1) cells (Figure 1B). In addition, the expression of the host receptor components (ACE2, TMPRSS2 and FURIN) required for SARS-CoV-2 cellular binding and fusion were also confirmed (Figure 1B). In vivo SARS-CoV-2 infections most likely occur at low a MOI and require multiple cycles of replication and infection for the virus to spread. Therefore, to mimic this process in vitro, the ALI cultures were infected apically with SARS-CoV-2 at a MOI of 0.1 and harvested as a function of time (24–96 h) post-infection (Figure 1A). The airway epithelium derived from each donor was readily infected with SARS-CoV-2, as indicated by staining for the viral nucleocapsid protein in the cultures 96 h post-infection (Figure 1C). The quantification of the number of nucleocapsid-positive cells at 96 h post-infection revealed that, on average, 8.4% (±1.9%) of the cells were infected with SARS-CoV-2 (Figure 1C). As a surrogate marker for virus replication, we quantified the expression of the nucleocapsid gene as a function of time post-infection (Figure 1D). Despite differences in the magnitude of the expressions between donors, we observed a consistent trend of increasing nucleocapsid expression from 24–96 h post-infection. Next, we quantified the infectious virus production from the independent ALI wells at the same time points. Consistent with the previous studies [18,23], we were unable to detect the infectious virus released into the basolateral chamber at any time point (data not shown). However, the infectious virus was readily detected in the apical chamber for each donor culture starting at 24 h post-infection. In contrast to the nucleocapsid gene expression, the infectious virus production demonstrated a high degree of variability at the later stages of infection (i.e., 96 h), with donor-specific trends (Figure 1E). For Donors 1–3, the peak virus release occurred at 48 h post-infection, with either a subsequent decrease or plateauing at 72 h and 96 h. The infectious virus release for Donor 2 at 96 h post-infection was below the limits of detection. In contrast to Donors 1–3, the SARS-CoV-2 infection of Donor 4 led to an increasing virus production from 48 to 96 h post-infection (Figure 1E). In summary, our data demonstrated that our ALI-differentiated airway epithelium generated using HBECs isolated from aged individuals are permissive of SARS-CoV-2 infection.

### 3.2. SARS-CoV-2 Infection Induces a Proinflammatory and Interferon Response in HBECs from Aged Individuals

**Figure 2:**
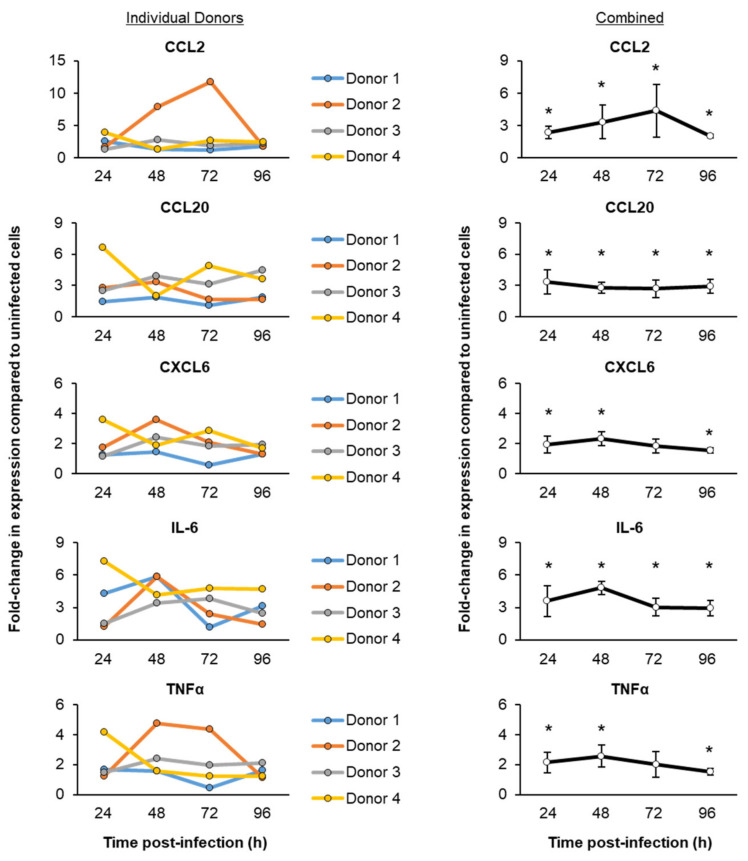
Temporal kinetics of the host proinflammatory cytokine and chemokine responses following SARS-CoV-2 infection. The primary HBECs (*n* = 4 donors) were cultured on ALI for 29–31 days, then infected with SARS-CoV-2 at a MOI of 0.1. The cells were then harvested as a function of time post-infection (24–96 h) for a qPCR analysis of the immune related genes CCL2, CCL20, CXCL6, IL-6 and TNFα. In the left panel, the data are presented for each individual donor, with each data point representing the mean fold-change in expression compared to the uninfected cells from *n* = 3 ALI wells. In the right panel, the data are combined and presented as the mean fold-change in expression compared to the uninfected cells from *n* = 4 donors. The error bars indicate the SEM. * *p* < 0.05.

**Figure 3:**
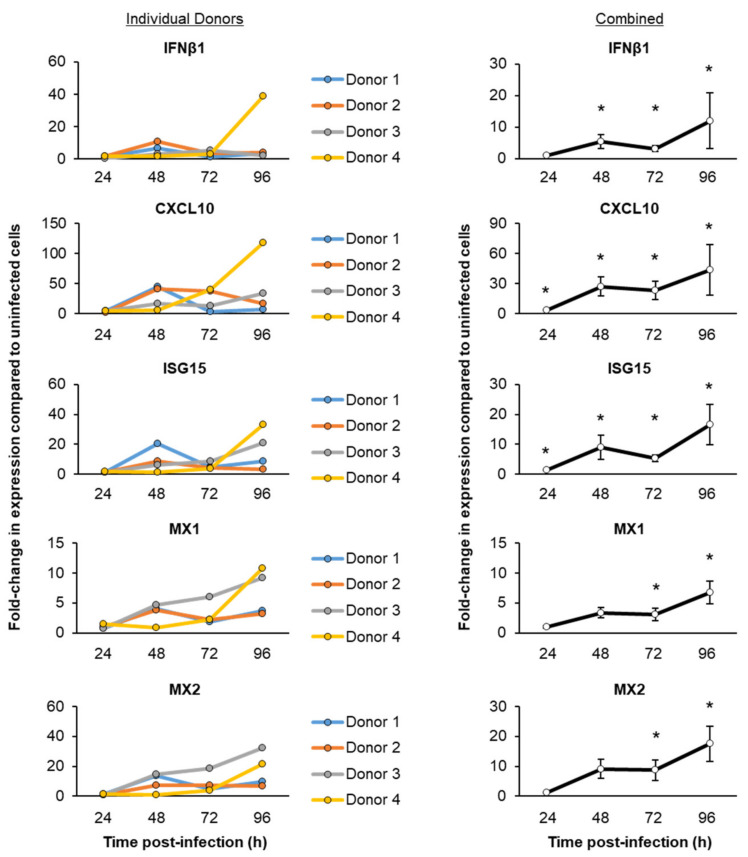
Temporal kinetics of the host interferon response following the SARS-CoV-2 infection. The primary HBECs (*n* = 4 donors) were cultured on the ALI for 29–31 days, then infected with SARS-CoV-2 at a MOI of 0.1. The cells were then harvested as a function of time post-infection (24–96 h) for a qPCR analysis of the interferon-related genes IFNβ1, CXCL10, ISG15, MX1 and MX2. In the left panel, the data are presented for each individual donor, with each data point representing the mean fold-change in expression compared to the uninfected cells from *n* = 3 ALI wells. In the right panel, the data are combined and presented as the mean fold-change in expression compared to the uninfected cells from *n* = 4 donors. The error bars indicate the SEM. * *p* < 0.05.

To evaluate the innate immune response to a SARS-CoV-2 infection, we next performed a targeted qPCR analysis of the proinflammatory cytokines, chemokines and interferon response genes previously associated with SARS-CoV-2 infection and COVID-19 (Figure 2) [6,10,16,18,19,23,35,36,37]. Despite the differences in the magnitude of induction between each HBEC donor, combined, we observed a consistent trend of proinflammatory cytokine and chemokine (CCL2, CCL20, CXCL6, IL-6 and TNFα) inductions within 24 h post-infection with SARS-CoV-2 (Figure 2). However, the interferon response (IFNβ1, CXCL10, ISG15, MX1 and MX2) was delayed and increased at 48 h post-infection (Figure 3).

**Figure 4:**
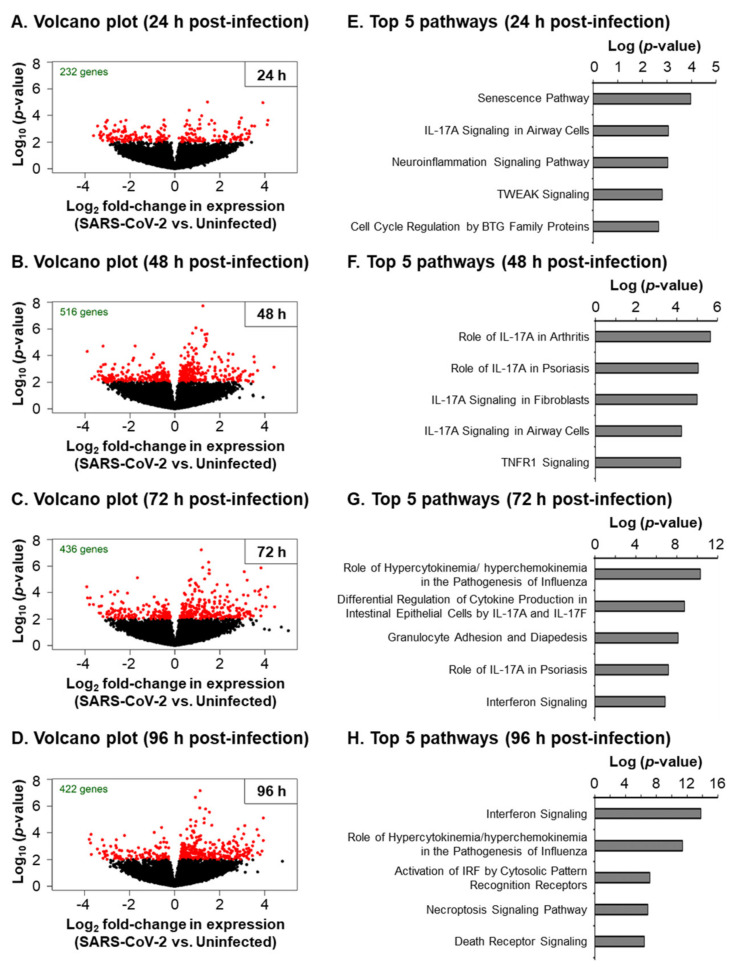
The bulk RNA-Seq analysis of the SARS-CoV-2-infected primary HBEC cultures from aged donors. (**A**–**H**) The primary HBECs (*n* = 4 donors) were cultured on the ALI for 29–31 days, then infected with SARS-CoV-2 at a MOI of 0.1. The cells were then harvested as a function of time (24–96 h) post-infection for the bulk RNA-Seq. (**A**–**D**) For each time point, the volcano plots represent the significantly (*p* < 0.01) differentially expressed genes (DEGs) between SARS-CoV-2 vs. the Uninfected cells. (**E**–**H**) The pathways enriched in the DEG list for each time point were identified by the Ingenuity Pathway Analysis (IPA), with the top five enriched pathways based on the *p*-value presented.

To further characterize the host response to SARS-CoV-2 infection, we performed a bulk RNA-Seq analysis on our uninfected and infected cultures at the same time points (Figure 1A). Using a significance threshold of a FDR < 0.05 (false discovery rate multiple testing adjusted *p*-value), we identified a total of *n* = 35 differentially expressed genes (DEGs) across all the time points (Supplementary File S1). However, to increase the testing power in the follow-up functional gene set discovery, we decreased our statistical stringency (*p*-value < 0.01), which allowed for identifying a larger number of DEGs (Figure 4 and Supplementary File S1). The number of significant DEGs more than doubled between 24 h (232 genes) and 48 h (516 genes) post-infection (Figure 4A,B). However, the number of significant DEGs were reduced at 72 h (436 genes) and plateaued at 96 h (422 genes) post-infection (Figure 4C,D). An Ingenuity Pathway Analysis of the DEGs for each time point demonstrated the enrichment of the molecular pathways associated with a robust innate immune response (Figure 4E–H and Supplementary File S1).

**Figure 5:**
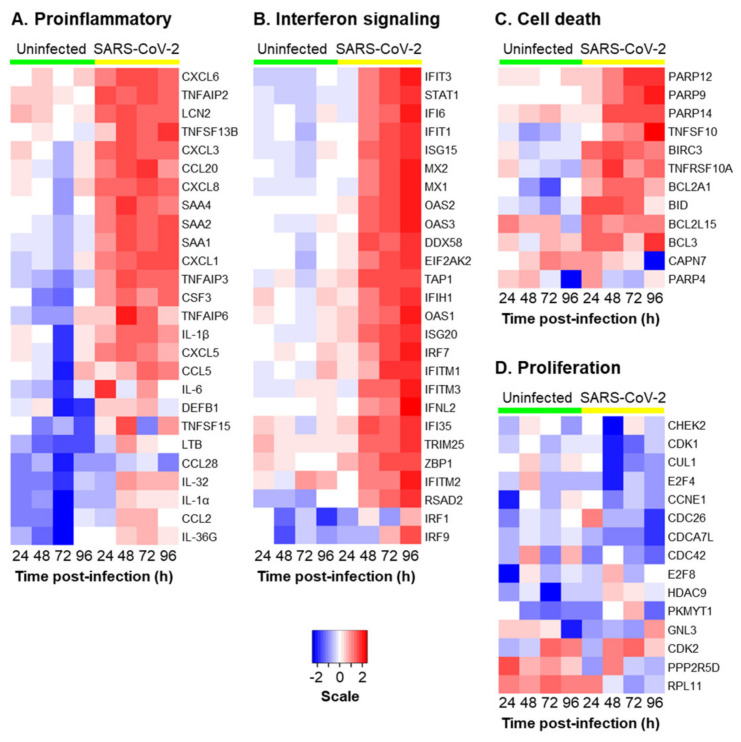
The SARS-CoV-2 infection of the primary HBEC cultures from aged donors induces a robust innate immune and host response. The heatmaps show the temporal expressions of example significantly (*p* < 0.01) differentially expressed genes (DEGs) identified by the bulk RNA-Seq between the SARS-CoV-2 vs. Uninfected cells as a function of time post-infection (24–96 h). The DEGs are grouped based on the biological functions. (**A**) Proinflammatory molecules. (**B**) Interferon signaling. (**C**) Cell death. (**D**) Proliferation. Scale bar represents the log fold-change in the expression relative to time 0 rescaled by the gene.

**Figure 6:**
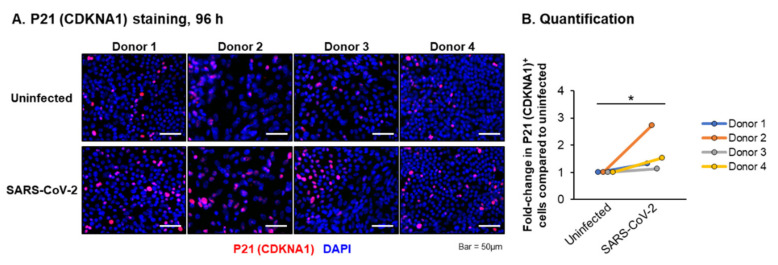
The SARS-CoV-2 infection of the primary HBEC cultures from aged donors leads to an increase in the number of P21 (CDKNA1)-positive cells. (**A**) The primary HBECs (*n* = 4 donors) were cultured on ALI for 29–31 days, then infected with SARS-CoV-2 at a MOI of 0.1 and then fixed 96 h post-infection. Immunofluorescent staining of P21 (CDKNA1) (red) and the nuclei (blue, DAPI) in uninfected and SARS-CoV-2-infected cells for each donor. Scale bar = 50 µm. (**B**) The quantification of P21 (CDKNA1)-positive cells. The data is presented for each donor as a fold-change in the positive cell number compared to the uninfected cultures. * *p* < 0.05.

Consistent with our targeted qPCR analysis (Figure 2 and Figure 3), we observed an enrichment of the pathways related to inflammation (e.g., IL-17 Signaling, the Neuroinflammation Signaling Pathway, Tumor Necrosis Factor Receptor (TNFR) Signaling and NF-κB Signaling) between 24 and 72 h post-infection (Figure 4E–G and Supplementary File S1), whereas, at 72 h and 96 h post-infection, we observed an enrichment of the pathways related to the interferon response and viral infection (e.g., Interferon Signaling, the Role of Hypercytokinemia/hyperchemokinemia in the Pathogenesis of Influenza, Activation of the Interferon Regulatory Factor (IRF) by Cytosolic Pattern Recognition Receptors and the Role of RIG1-like Receptors in Antiviral Innate Immunity) (Figure 4G,H and Supplementary File S1). In addition to the innate immune response pathways, we observed the enrichment of the pathways related to cell death and senescence throughout the infection (e.g., the Necroptosis Signaling Pathway, Death Signaling, Retinoic acid Mediated Apoptosis Signaling and Senescence Pathway) (Figure 4 and Supplementary File S1). Recent studies have suggested that epigenetic regulation (e.g., miRNAs, DNA and RNA methylation, histone acetylation and methylation) and the host oxidative stress response play important roles in regulating the outcome of SARS-CoV-2 infection [53,54,55,56]. While we did not observe a significant enrichment of these pathways in response to the SARS-CoV-2 infection, we did observe changes in the expression of individual genes associated with each of these pathways (Supplementary Figures S1–S3). To better visualize the expression kinetics of the genes associated with each of these pathways and biological processes significantly enriched in response to SARS-Cov-2, heatmaps were generated for the significant DEGs identified in our study. Due to the presence of some DEGs in multiple pathways, we grouped the genes into the following four categories: “Proinflammatory molecules”, “Interferon signaling”, “Cell death” and “Proliferation” (Figure 5). Consistent with the pathway analysis, SARS-CoV-2 infection led to the robust induction of multiple cytokines (e.g., IL-1α, IL-1β and IL-6) and chemokines (e.g., CCL2, CCL5 and CCL20) at 24 h post-infection (Figure 5A), whereas the induction of interferon signaling and expression of the downstream interferon signaling genes (ISGs) (e.g., DDX58, ISG15 and MX1) was delayed and apparent at 48 h post-infection (Figure 5B). The expression of cell death and apoptosis-associated genes (e.g., BID, BIRC3 and PARP14) increased throughout the infection, suggestive of increased cell death in response to the infection (Figure 5C). Furthermore, SARS-CoV-2 infection led to the modulation of multiple genes associated with senescent cells, including a downregulation of the proliferation-associated genes (e.g., CDK1, E2F4 and CDC42) (Figure 4D) and increased expression of the genes related to the senescence-associated secretory phenotype (SASP) (e.g., IL-1α, IL-6 and CXCL8) (Figure 5A). While we observed no significant changes in the expression of the cell cycle arrest genes in response to the virus infection, we did observe a trend of increased expression for RB1, TP53 and P21 (CDKN1A) at specific time points post-infection (Supplementary Figure S3B). To further characterize the senescence phenotype, we performed staining for the senescence marker P21 (CDKN1A) in the cultures 96 h post-infection (Figure 6A). The quantification revealed a small but significant increase (average 1.7-fold) in the number of P21 (CDKN1A)-positive cells in SARS-CoV-2-infected vs. uninfected cultures (Figure 6B). In summary, these findings indicate that the SARS-CoV-2 infection of differentiated HBEC cultures from aged donors leads to a potent host response, including the early induction of proinflammatory cytokines/chemokines, a delayed interferon response and modulation of the pathways that regulate cell death and senescence.

## 4. Discussion

An exaggerated immune response (i.e., “cytokine storm”) in response to SARS-CoV-2 infection plays a critical role in regulating COVID-19 disease progression and severity [10,34,35,36,37,38,39]. Therefore, to develop effective therapeutic strategies for the treatment of COVID-19, we first need to understand the host response to SARS-CoV-2 infection in the populations most vulnerable to disease. Advanced age (>60 years old) is the most significant risk factor for developing severe COVID-19, with these patients showing the highest rates of morbidity and mortality [43,45,46]. The mucociliary airway epithelium is the predominant site of the initial SARS-CoV-2 infection in vivo [1,5,6,7,8]. Therefore, in this study, we used in vitro ALI cultures of differentiated primary HBECs isolated from older individuals (>67 years old) to characterize the SARS-CoV-2 virus–host interactions in the “aged” mucociliary epithelium. Our study demonstrates that the ALI epithelial cultures established from older HBEC donors are permissive to SARS-CoV-2 infection, resulting in the production of an infectious virus and a potent host response enriched with pathways associated with epithelial innate immunity.

In accordance with other in vitro ALI studies [16,18], we observed a large variability in the kinetics and magnitude of the virus replication between HBEC donors. The same stock of virus was used for each experiment; therefore, this variability may result from differences in the expression levels of the host receptor components (e.g., ACE2) between the cultures, which may either limit or enhance the virus infection and spread. Alternatively, the cell intrinsic genetic factors specific to each donor may influence the virus replication post-entry. Despite the variability in the virus growth kinetics between the HBEC donors, we consistently observed a rapid proinflammatory response during the early stages (24 h) of SARS-CoV-2 infection characterized by an increased expression of multiple cytokines (e.g., IL-6) and chemokines (e.g., CCL2). The epithelial expression of these factors likely plays an important role in regulating the inflammatory response to viral infections in vivo via the regulation of the localized barrier function and recruitment of immune cells. Indeed, elevated levels of CCL2, CXCL10, IL-6 and TNFα have been identified in severe COVID-19 patients compared to patients with a mild form of the disease [35,37,39]. In addition to the proinflammatory response, our data show that SARS-CoV-2 infection of the mucociliary airway epithelium results in a delayed induction of the types I and II IFN responses characterized by the expression of multiple interferon-stimulated genes (ISGs). This delay may, in part, be due to the expression of various SAR-CoV-2 proteins that suppress the cellular IFN responses to perturb the host innate immune response [1,14]. The treatment of cells with types I and III IFNs can suppress SARS-Cov-2 replication in the airway epithelium [16,23]. Therefore, the successful induction of IFN signaling following virus infection may explain why we observed low numbers of infected cells in our cultures at 96 h post-infection. While our findings that SARS-CoV-2 infection results in a delayed IFN response are consistent with multiple studies [16,18,19], some studies have shown that SARS-CoV-2 infection leads to either a lack of or a low level of induction of the IFN response [10,23]. This discrepancy in IFN induction most likely reflects the technical differences between each study, including the time points of the analyses post-infection (i.e., early vs. late) and the virus MOI used.

The induction of apoptosis has been reported as a feature of the SARS-CoV-2 infection of lung epithelial cells [15,16,18,26]. In support of this, we observed the enrichment of the pathways related to this process in response to a virus infection in our ALI cultures. In combination with the potent proinflammatory response following the virus infection, increased levels of apoptosis may play an important role in driving the lung epithelial tissue damage and immune pathogenesis associated with SARS-CoV-2 infection and the development of severe COVID-19 [34,57]. One interesting and novel observation from our study was the enrichment of the “Senescence Pathway” in response to the virus infection, which was characterized by the altered expression of multiple senescence-associated genes and increased numbers of P21 (CDKNA1)-positive cells. Cellular senescence is characterized as a state of stable growth arrest, resistance to apoptosis and adoption of the senescence-associated secretory phenotype (SASP) [58]. Via the secretion of multiple SASP factors related to the immune functions (e.g., IL-1β, IL-6 and IFNs) and tissue remodeling (e.g., TGFβ family ligands), senescent cells play an important role in regulating the tissue homeostasis, regeneration and repair [58]. The number of senescent cells accumulate with age and are associated with the development of multiple chronic diseases, including in the lungs [59,60,61]. Furthermore, the induction of senescence in response to a virus infection can occur via a direct infection or in a paracrine manner via the prolonged exposure of cells to proinflammatory cytokines and IFNs released following activation of the innate immune response [62,63]. Based on the knowledge that SARS-CoV-2 induces apoptosis in infected cells [15,16,18,26], we hypothesized that SARS-CoV-2 infection induces senescence of the airway epithelial cells in a paracrine manner. Aged cells are more susceptible to undergo senescence in response to environmental insult, which may explain why we observed the enrichment of the senescence pathways compared to other studies that used similar experimental models [16,18,19,23]. Therefore, the “cytokine storm” induced in response to the SARS-CoV-2 infection may have enhanced the senescent-inducing effects in older vs. young patients, which may play a role in promoting an increased COVID-19 disease morbidity and mortality in older populations [64]. Despite our findings, future studies are required to investigate the mechanisms of how a SARS-CoV-2 infection leads to the induction of cellular senescence and the role of senescent cells in the pathogenesis of SARS-CoV-2 and COVID-19 disease severity.

In summary, our in vitro ALI model of differentiated human mucociliary airway epithelium provided an important system to characterize the temporal kinetics of the host response to a SARS-CoV-2 infection in the airway epithelium of older individuals. The limitations for our study include the small number of primary HBEC donors and the lack of donors from other age groups (i.e., young and middle-aged individuals), which prevented us from to identifying the “age-dependent” host responses to SARS-CoV-2 infection. Furthermore, the use of bulk vs. single-cell RNA-Seq approaches prevented us from characterizing the responses of individual epithelial cell types to the virus infection. Finally, our model lacked the presence of in vivo lung resident microbial flora and inflammatory cells, which may interact with the airway epithelium to modulate the local immune response to virus infections. Despite these limitations, by assessing the transcriptome of the airway epithelial cells from the most vulnerable population infected with SARS-CoV-2, we showed that a virus infection leads to the potent induction of cellular pathways linked to an innate immunity, inflammation, cell death and senescence. Future studies will focus on studying the role of these pathways in regulating SARS-CoV-2 infection and replication both in vitro and in vivo.

## Data Availability

The raw data from the bulk RNA-Seq studies are publicly available at the Gene Expression Omnibus (GEO) site (http://www.ncbi.nlm.nih.gov/geo/), accession number GSE175779.

## Supplementary Materials

The following are available online at https://www.mdpi.com/article/10.3390/v13081603/s1: Supplementary Figure S1. Heatmaps showing temporal expression of genes related to miRNA biogenesis, DNA and RNA methylation identified by bulk RNA-Seq between SARS-CoV-2 vs. Uninfected cells (*n* = 4 donors of primary HBECs) as a function of time post-infection (24–96 h). The genes have been grouped based on biological function. (**A**) miRNA biogenesis. (**B**) DNA demethylases and methyltransferases. (**C**) RNA methyltransferases. Scale bar represents log fold change in expression relative to time 0, rescaled by gene. Supplementary Figure S2. Heatmaps showing temporal expression of genes related to histone acetylation and methylation identified by bulk RNA-Seq between SARS-CoV-2 vs. Uninfected cells (*n* = 4 donors of primary HBECs) as a function of time post-infection (24-96 h). The genes have been grouped based on biological function. (**A**) Histone acetylases. (**B**) Histone deacetylases. (**C**) Histone methyltransferases. Scale bar represents log fold change in expression relative to time 0, rescaled by gene. Supplementary Figure S3. Heatmaps showing temporal expression of genes related to oxidative stress and cell cycle arrest by bulk RNA-Seq between SARS-CoV-2 vs. Uninfected cells (*n* = 4 donors of primary HBECs) as a function of time post-infection (24–96 h). The genes have been grouped based on biological function. (**A**) Oxidative stress. (**B**) Cell cycle arrest. Scale bar represents log fold change in expression relative to time 0, rescaled by gene. Supplementary File S1. The differentially expressed genes (DEGs) and molecular pathways altered in response to the SARS-CoV-2 infection identified by the bulk RNA-Seq.
