# Supplementary material for: Characterization of the SARS-CoV-2 Host Response in Primary Human Airway Epithelial Cells from Aged Individuals
Source: Viruses. 2021 Aug 12;13(8):1603. doi: 10.3390/v13081603 (PMC8402710; doi:10.3390/v13081603)
Supplement: Supplementary file 1 [file viruses-13-01603-s001.zip › suppl for virus....092/viruses-1276092 rev suppl2.pdf]

Supplemental Figure 1

A. miRNA biogenesis

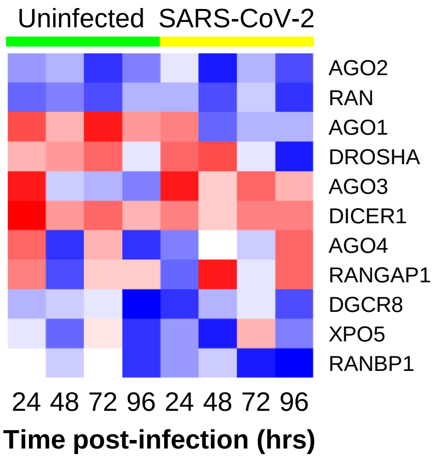

B. DNA demethylases and methyltransferases

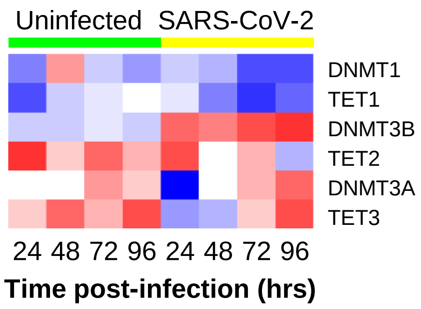

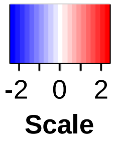

C. RNA methyltransferases

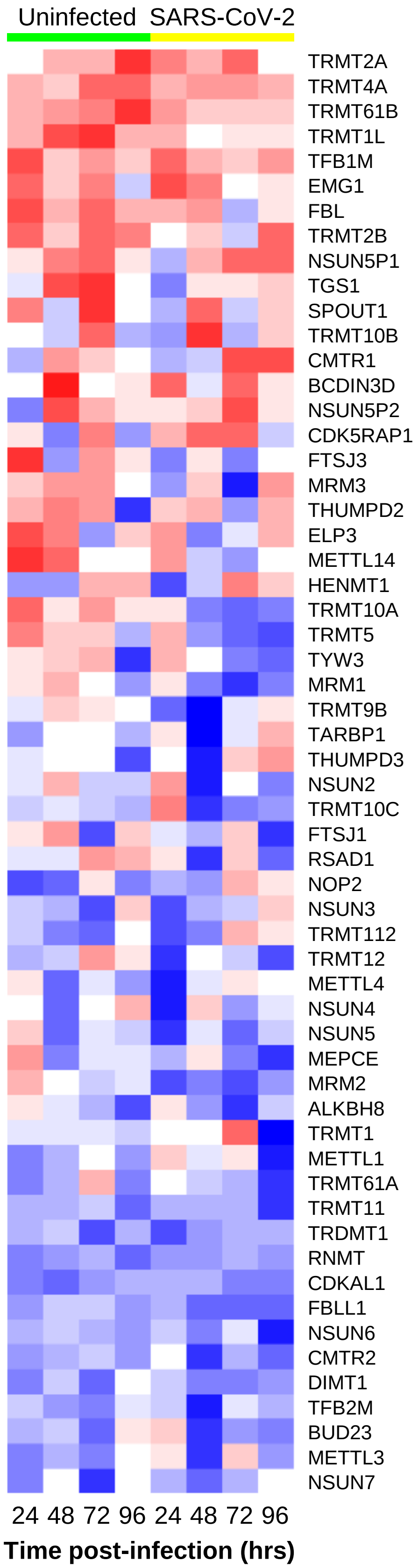

Supplementary Figure 1. Heatmaps showing temporal expression of genes related to miRNA biogenesis, DNA and RNA methylation identified by bulk RNA-Seq between SARS-CoV-2 *vs* Uninfected cells (n=4 donors of primary HBECs) as a function of time post-infection (24-96 hrs). The genes have been grouped based on biological function. (**A**) miRNA biogenesis. (**B**) DNA demethylases and methyltransferases. (**C**) RNA methyltransferases. Scale bar represents log fold change in expression relative to time 0, rescaled by gene.

Supplemental Figure 2

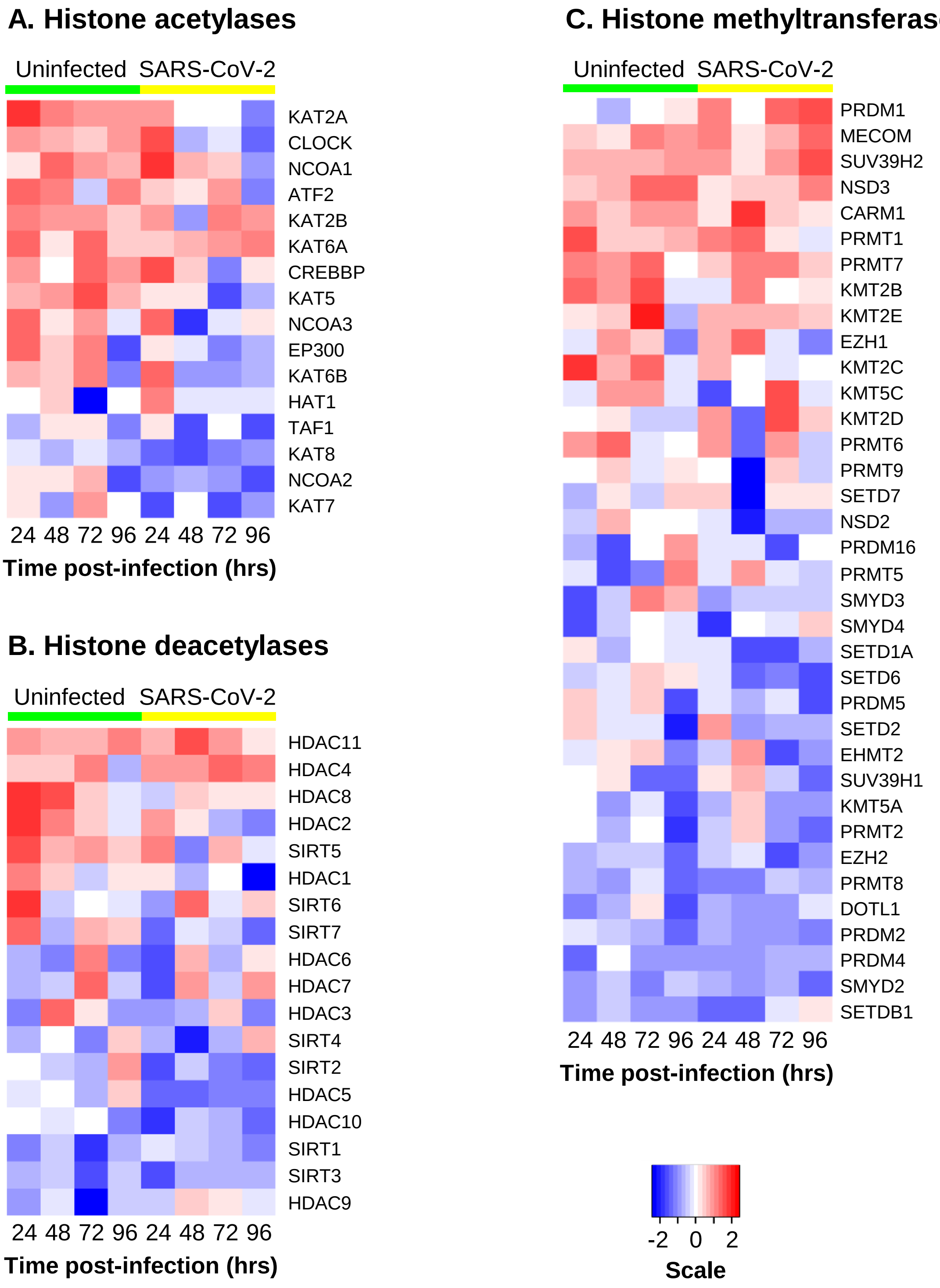

Supplementary Figure 2. Heatmaps showing temporal expression of genes related to histone acetylation and methylation identified by bulk RNA-Seq between SARS-CoV-2 *vs* Uninfected cells (n=4 donors of primary HBECs) as a function of time post-infection (24-96 hrs). The genes have been grouped based on biological function. (**A**) Histone acetylases. (**B**) Histone deacetylases. (**C**) Histone methyltransferases. Scale bar represents log fold change in expression relative to time 0, rescaled by gene.

Supplemental Figure 3

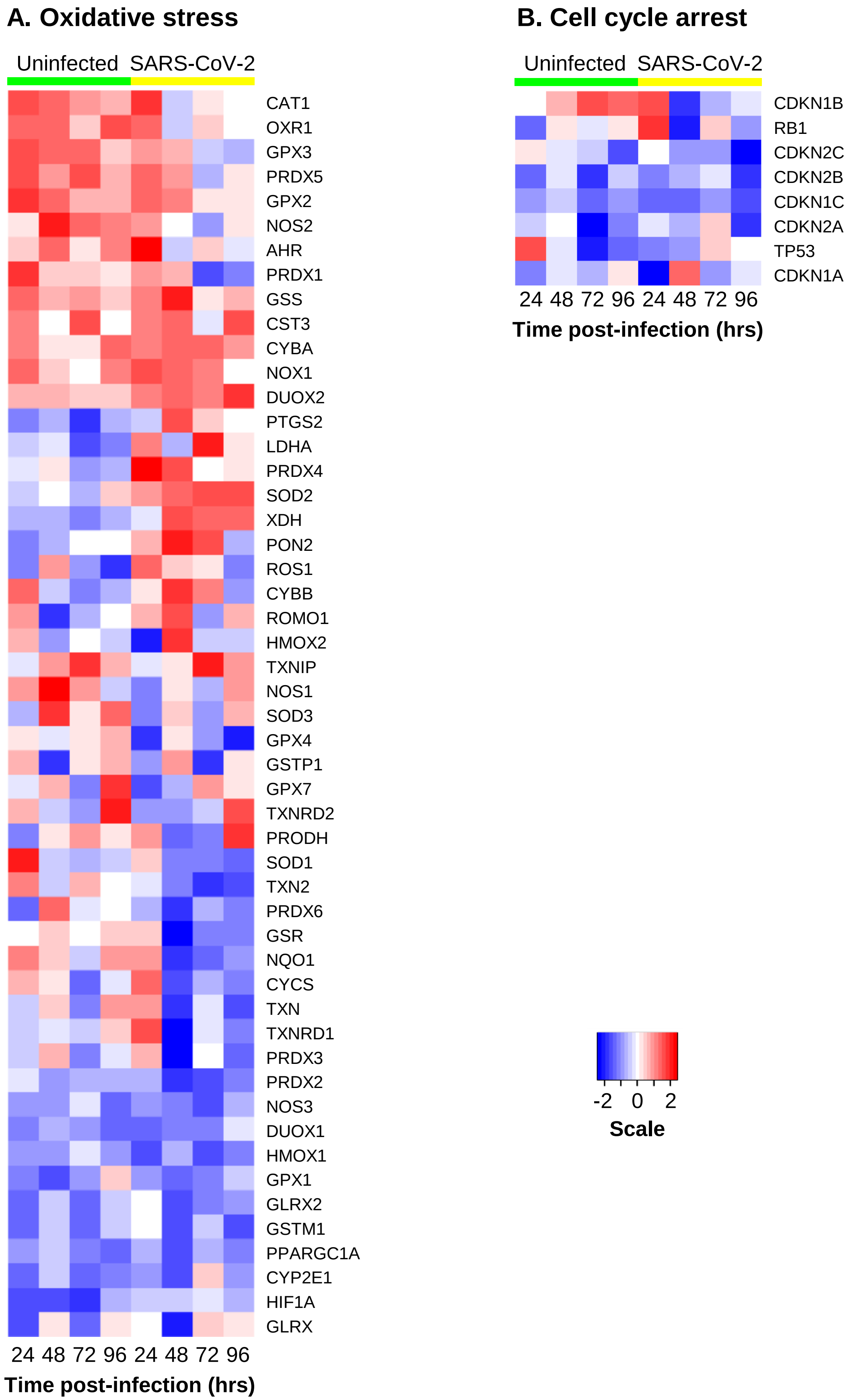

Supplementary Figure 3. Heatmaps showing temporal expression of genes related to oxidative stress and cell cycle arrest by bulk RNA-Seq between SARS-CoV-2 *vs* Uninfected cells (n=4 donors of primary HBECs) as a function of time post-infection (24-96 hrs). The genes have been grouped based on biological function. (**A**) Oxidative stress. (**B**) Cell cycle arrest. Scale bar represents log fold change in expression relative to time 0, rescaled by gene.
